# Mediating Role of Perceived Stress in the Relationship between Facing Existential Issues and Symptoms of Depression and Anxiety 

**Published:** 2020-01

**Authors:** Mohammad-Ali Besharat, Hossein Khadem, Vahid Zarei, Ali Momtaz

**Affiliations:** 1 Department of Psychology, School of Psychology and Educational Sciences, University of Tehran, Tehran, Iran.; 2 Department of Psychology, School of Psychology and Educational Sciences, Shahid Beheshti University, Tehran, Iran.; 3 Department of Psychology, School of Psychology and Educational Sciences, Ferdowsi University, Mashhad, Iran.; 4 Department of psychology, School of Psychology and Educational Sciences, Semnan University, Semnan, Iran.

**Keywords:** *Anxiety Symptoms*, *Depression Symptoms*, *Death*, *Freedom*, *Loneliness*, *Meaningfulness*, *Perceived Stress*

## Abstract

**Objective:** This study aimed at investigating the mediating role of perceived stress in anticipation of anxiety and depression on facing the ultimate concerns (death, loneliness, freedom, and meaningfulness).

**Method** **:** A total of 389 students from Ferdowsi University of Mashhad participated in this study in 2017-18. The participants were selected using random sampling. The data were collected using the subscales of anxiety and depression in depression, anxiety, stress scale (DASS), Death Anxiety Scale (DAS), the third edition of Loneliness Scale UCLA (UCLA-LS), the subscale of meaning in life in Meaningfulness of Life Questionnaire (MLQ), responsibility scale of California Psychological Inventory (CPI), and the Perceived Stress Scale (PSS). Then, they were evaluated using Pearson correlation and path analysis.

**Results: **The correlation between the symptoms of anxiety and depression, and death anxiety and loneliness was direct and significant with the perceived stress. The relationship between the perceived stress with meaningfulness of life and responsibility was significantly inverse. The analysis of the data path showed that the component dealing with existence (loneliness and death anxiety) predicted 20% of anxiety symptoms through perceived stress in the model that was fitted well with research data.

**Conclusion: **According to the findings, it can be concluded that the relationship between dealing with ultimate concerns and anxiety symptoms was not linear. Dealing with ultimate concerns affects the anxiety symptoms through the perceived stress. Therefore, attention to the perceived stress management to promote health and prevent anxiety disorders is important.

In contrast to fear, the anxiety response pattern is a complex blend of unpleasant emotions and cognitions that is both more oriented to the future and much more diffuse than fear. However, like fear, it has not only cognitive/subjective components but also physiological and behavioral components (1). The overall prevalence of at least one anxiety disorder in the 12-month period nationwide (Iran) is more than many countries. In the national survey of mental health, the prevalence of these disorders in Iran was 15.6% (2). Depression is a serious emotional disturbance and a significant health problem (3). The overall prevalence of depression disorders in Iran is 12.7% (2). 

Moreover, anxiety and depression disorders have common factors, including emotional basis, comorbidity and overlap for identification, limitation in decision-making, anxiety, rumination, perfectionism, obsessions, and repeated and common biological deficiencies in limbic system (1, 3). Thus, they have many common risk factors. Anxiety and depression can be caused by psychosocial, physical, and biological aspects, medical disorders, medication side effects or a combination of all these factors. Psychosocial factors include internal states of the individual, unconscious motivations, and the individual's inability to cope with stress, such as work, marriage, occupational hazards, separation, rejection, loss, etc. (4, 5). 

Each of these situations can also act as an occasion to meet the ultimate concerns. Thus, the individual is faced with the ultimate concerns, including death, loneliness (or being isolated), freedom (or responsibility in life), and meaninglessness. This confrontation can act as a risk factor for developing anxiety and depression (6,7).

According to this view, existential psychologists consider the fear of death, lack of meaning in life, burden of freedom (or responsibility in life), and pain of loneliness as fundamental causes of various disorders, especially anxiety and depression (7, 8, 9, 10). In other words, death, loneliness (or being isolated), meaninglessness, and freedom (or responsibility in life) are 4 ultimate concerns. Yalom (1980) proposed that dealing with these certainties are 4 ultimate concerns for humans. He considered the inability to healthy confrontation with these certainties or existential issues as the reason for many mental abnormalities (7). For example, in a research that examined the procedure of meaning in amyotrophic lateral sclerosis (ALS), patients experienced many anxiety-filled days in the life and death anxiety. This study also reported experiences such as meaninglessness, shame, guilt, existential loneliness, injustice, and disability in those patients (11). However, some patients find meaning of life through means such as friends and family, giving and receiving help, and acceptance of the present time. Thus, these patients experience less anxiety symptoms and death anxiety with increasing meaning in life. With respect to depression and the components dealing with the ultimate concerns, one-third of depressed patients who have treatment-resistant depression can be noted (12,13). On the other hand, Stålsett, G., Gude, T., Rønnestad, M., and H., & Monsen, (2012) reported experiences such as religion, loneliness, meaningless, death, loss of freedom, fear of freedom and responsibility, guilt, and shame in patients with depression who have treatment-resistant depression (14).

One of the questions that researchers consider on the relationship between dealing with the ultimate concerns and symptoms of anxiety and depression is the linear or nonlinear nature of this relationship. The following findings pose the assumption that the perceived stress can have a decisive role in dealing with the ultimate concerns and symptoms of anxiety and depression.

Stress is evoked in people when they perceived themselves disabled against their demands or their well-being threats (15). Therefore, the effect of stressful events depends on perception of the stress (16). Cohen, S., and Kamarck, T., & Mermelstein (1983) consider the perceived stress as an appropriate construct for the relationship between stress and pathology (17). Stress is one of the risk factors for anxiety disorders and depression (18, 19). In patients with generalized anxiety disorder and major depression, sensitivity to stress is higher than normal individuals (18, 20 and 21). On the other hand, stress has 2 elements: (1) the lack of comfort and (2) pressure. People often complain of pressure, but the problematic element of stress is a lack of comfort. In fact, stress reveals a variety of acute crisis, which confirms lack of comfort in individuals. Crises in life can force one to face existential issues (22, 23). In a qualitative study among 15 nurses in Sweden, stress was correlated with existential dimensions (24). 

Prior studies have shown that facing existential issues has an effect on perceived stress, anxiety, and depression. Anxiety and depression were influenced by perceived stress. However, investigating the role of perceived stress in the relationship of anxiety and depression with existential issues is a subject not tackled in the literature so far. Thus, this study was conducted to address this gap by investigating the mediating role of perceived stress in the prediction of anxiety and depression based on facing existential issues. Moreover, this study investigated the relationship between anxiety, depression, and existential issues with perceived stress. Figure 1 displays the hypothetical model of this study.

## Materials and Methods

***Population, Sample, and Sample Selection Method***

This study was conducted in Ferdowsi University of Mashhad in 2017-18. According to the Kerjsy – Morgan Table (25), 378 students were selected as the sample size. However, the researchers studied 400 people because of the possibility of distortion of some of questionnaires and possibility of sampling error. Moreover, to reduce the sampling and non sampling error, questionnaires were provided in 2 different orders to control the effects of fatigue and the orders. Therefore, one order of questionnaires was DASS, DAS, UCLA-LS, MLQ, CPI, and PSS, and the other order was MLQ, CPI, PSS, DASS, DAS, and UCLA-LS. The inclusion criteria were willingness to participate in study and studying at Ferdowsi University of Mashhad. The age range of participants was 18-30 years. After visiting all faculties of Ferdowsi University of Mashhad, the students volunteered to participate in the research. Then, they were provided with the necessary explanations about the purpose of research. Also, students’ anonymity was preserved. The exclusion criteria were incomplete questionnaires and special arrangements for answering the questionnaire. Moreover, the presence and history of psychotic disorders was investigated and those students with such diseases were excluded from the study. Based on the exclusion criteria, 11 students were excluded and the number of sample was reduced to 389 students. All of the rest of questionnaires were coded by a number and kept anonymous. The approximate time to fill out the questionnaire was 30 minutes, during which the participants completed depression, anxiety, stress scale (DASS), Death Anxiety Scale (DAS), the third edition of Loneliness Scale UCLA (UCLA-LS), the subscale of meaning in life in Meaningfulness of Life Questionnaire (MLQ), responsibility scale of California Psychological Inventory (CPI), and the Perceived Stress Scale (PSS). Data were analyzed using SPSS version 23.0 and lisrel software version 9.2. The analyzing methods were Pearson correlation coefficients and structural equation model (SEM).

***Measuring Tool***

- **Depression, Anxiety, Stress Scale (DASS):** This scale was developed by Lovibond and Lovibond (1995). This 21-item test measures the symptoms of depression, anxiety, and stress. Also, it is a valid instrument for assessing symptoms of negative emotions and its reliability and validity have been confirmed in multiple studies (26-30). The Cronbach's alpha coefficients of the DASS subscales and the total number of DASS for depression, anxiety, stress, and the total number were, respectively, 0.87, 0.85, 0.89, and 0.91. The concurrent, convergent, and discriminant validity of the Depression Anxiety Stress Scale were calculated and approved through the simultaneous implementation of Beck Depression, Beck Anxiety Scale, Positive and Negative Affect Schedule, and Mental Health Inventory (27).
- **Death Anxiety Scale:** Templer developed this scale at the University of Kentucky with 15 yes/no questions in which yes represents death anxiety. In the American society, the retest reliability coefficient of this scale has been reported to be 0.83; its concurrent validity has been reported to be 0.27, with the Manifest Anxiety Scale, and 0.40, with Beck Depression scale (p<0.001) (31). To assess the convergent validity of the scale, its correlation was reported to be 0.43, with the scale of anxiety (p < 0.001) (32). Tavakoli and Ahmadzadeh (2011) also found the reliability of this test through test-retest, split-half, and Cronbach's alpha coefficient to be 0.87, 0.59, and 0.75, respectively (33).
- **Loneliness Scale UCLA (Third Edition): **This scale was first made in 1978 by Russell, Peplau, and Ferguson at the University of California. The reliability of the scale using Cronbach's alpha coefficient has been reported from 0.89 to 0.94. The convergent validity of the scale was demonstrated by significant correlations with other tests (34). The Cronbach's alpha coefficient in an Iranian society was 0.89. The correlation coefficient between this scale and the loneliness score is changing from the total scale from 0.261 to 0.636 (p < 0.001), which indicates a good internal validity scale. In addition, the divergent validity of this scale through its correlation is 0.67 with Beck Depression Inventory (p < 0.01) (35).
- **Meaning of Life Questionnaire**: This scale was presented by Steger, Frazier, Ishii, and Kaller (2006) to assess the meaning of life. The validity, reliability, and factor structure have been analyzed in different studies with different samples. Meaning of Life Scale includes 2 subscales which assess the meaning of life and search for higher meanings. The Cronbach's alpha reliability of finding meaning and searching for meaning subscales is 0.70 and 0.73, respectively. Convergent validity of the scale is 0.61 to 0.74 (p < 0.001) through correlation with similar subscales (36). The test-retest reliability of this scale in Iran with a 2-week interval is 0.84 for the existence of meaning and 0.74 for searching for meaning subscales (p < 0.001). Cronbach's alpha was 0.75 for the existence of meaning and 0.78 for having meaning (37).
- **California Psychological Inventory Scale of Responsibility: **California Psychological Inventory is a self-implementation test that Gough designed in 1948 to identify the characteristics of a stable personality in healthy populations. The responsibility scale has 42 questions with 2 I agree and I disagree options. This scale assesses features such as conscientiousness, responsibility, trustworthiness, acting in accordance with discipline and believing that the reason must prevail over life. The questionnaire reliability coefficient is 0.70 (p < 0.001). The internal consistency coefficients of this questionnaire are from 0.52 to 0.82 (38,39). The Cronbach's alpha reliability of this scale in Iran in 2 different researches are 0.57 and 0.76. Test-retest reliability coefficient of this scale in a period of 2 weeks is 0.73 (p < 0.001) (40, 41).
- **Perceived Stress Scale:** This scale was developed by Cohen et al in 1983 with 14 questions with 5-point Likert scale (from none to high) and it measures perceived stress. Cronbach's alpha coefficients of the scale in 3 different studies were 0.84, 0.85, and 0.86, respectively. Cohen et al assessed the convergent validity of the scale through the simultaneous implementation of event-life scores and reported the correlation coefficients in different studies to be in the range of 0.14 to 0.76 (p < 0.001). Therefore, the convergent validity of the test is confirmed (17). Safai and Shokri (2014) reported the Cronbach's alpha coefficient of this scale to be 0.76 in a group of cancer patients (42).

## Results

A total of 218 men and 171 women participated in this study. The mean age of male and female participants was 20.84 ± 2.91 and 22.31 ± 3.67 years, respectively. The mean age of all the participants was 21.48 ± 3.33. Table 1 shows the mean and standard deviation of scores for symptoms of anxiety and depression, dealing with the existential issues and the perceived stress according to gender and for the entire sample of students in the academic year of 2017-18 at Ferdowsi University of Mashhad.

According to Table 2, the Pearson correlation coefficients showed that the correlation between anxiety and depression with the perceived stress was direct and significant. The relationship between perceived stress with death anxiety and loneliness was also direct and significant. However, the relationship between perceived stress with meaningfulness of life and responsibility was significant and inverse.

**Figure 1:**
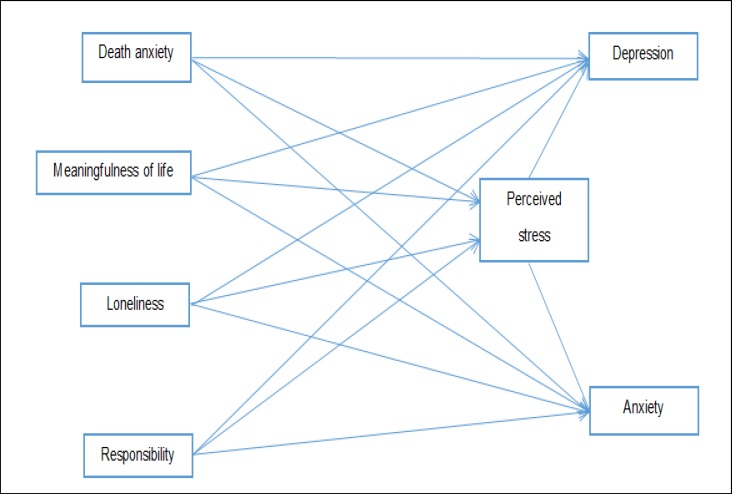
The Hypothetical Model of the Mediating Role of Perceived Stress in Anticipation of Anxiety and Depression Based on Facing Existential Issues

The model presented in Figure 1 was used to investigate the mediating role of perceived stress in the prediction of anxiety and depression based on facing the existential issues. According to this model, it is assumed that facing the existential issues through perceived stress affects the symptoms of anxiety and depression. After proving assumptions for the path analysis (normal distribution of data, lack of outliers and linear relationships between variables), the maximum likelihood (ML) method was used to test the model.

Direct, indirect, and total effects and the coefficient of determination of the path of the model are presented in Table 3.

Indicators of goodness-of-fit the model of the research has a good fit (RMR=0/04, PGFI= 0/31, PNFI= 0/40, CFI= 0/95, IFI= 0/95, NNFI= 0/89, NFI= 0/94, AGFI= 0/93, GFI= 0/98, P-VALUE= 0/00, DF= 9, Chi-2= 29/27, RMSEA= 0/07).

**Figure 2:**
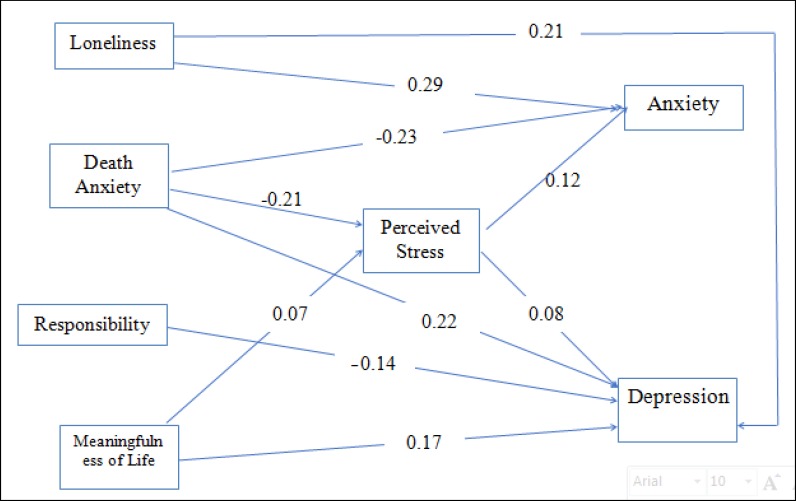
Indicators of Direct Effects in the Model (the Mediating Role of Perceived Stress in Predicting the Symptoms of Anxiety and Depression based on Loneliness, Meaningfulness of Life, Responsibility, and Death Anxiety)

Therefore, the mediating role of the perceived stress in the prediction of anxiety symptoms based on loneliness and death anxiety was confirmed, this model was showed in figure 2. Nevertheless, due to the lack of significant direct effect of the perceived stress on symptoms of depression, the mediating role of the perceived stress in the relationship between symptoms of depression and loneliness, meaningfulness of life, responsibility, and death anxiety was not confirmed.

**Table 1:** The Mean and Standard Deviation of Symptoms of Anxiety and Depression, Death Anxiety, Loneliness, Meaningfulness of Life, Responsibility, and Perceived Stress

**Variables and Indicators**	**Male Students**	**Female Students**		**All Students**	
	**Mean(SD)**	**Mean(SD)**	**Mean(SD)**	
Symptoms of Anxiety	4.22(4/04)	3.56 (3/76)	3.91(3/91)	
Symptoms of Depression	5.88(4/93)	5.14(4/85)	5.54 (4/89)	
Death Anxiety	7.57(3/02)	8.92(3/25)	8.16(3/18)	
Loneliness	46.43 (12/29)	40.24(10/74)	43.70 (12/00)	
Meaningfulness of Life	16.29(4/84)	16.68 (4/69)	16.46 (4/76)	
Responsibility	20.88 (4/64)	19.56(3/38)	20.30 (4/18)	
Perceived Stress	27.02 (7/69)	26.20 (7/98)	26.66(7/80)	

**Table 2:** Correlation Coefficient Matrix of Symptoms of Anxiety and Depression, Death Anxiety, Loneliness, Meaningfulness of Life, Responsibility, and Perceived Stress

**Variable**	**1**	**2**	**3**	**4**	**5**	**6**
1. Symptoms of Anxiety	1					
2. Symptoms of Depression	0.57**	1				
3. Death Anxiety	0.11*	0.11*	1			
4. Loneliness	0.30**	0.50**	0.09	1		
5. Meaningfulness of Life	-0.20**	-0.41**	-0.14**	-0.34**	1	
**6.** Responsibility	-0.15**	-0.08	0.10*	-0.01	0.20**	1
**7.** Perceived Stress	0.40**	0.51**	0.19**	0.36**	-0.37**	-0.15**

** P<0.01

* P<0.05

**Table 3:** Effects of the Mediating Role of Perceived Stress in the Relationship of Anxiety and Depression with Loneliness, Meaningfulness of Life, Responsibility, and Death Anxiety

**Paths **	**Direct Effect**	**Indirect Effect**	**Total Effect**	**Coefficient of Determination**
**Anxiety From:**				0.20
**Perceived Stress**	0.12**		0.12**	
**Death Anxiety **	-0.23**	0.02	-0.20**	
**Loneliness**	0.29**		0.29**	
**Meaningfulness of Life**	0	0.008	0.008	
**Depression From:**				0.19
**Perceived Stress**	-0.08		-0.08	
**Death Anxiety **	0.22**	0.01	0.23**	
**Loneliness**	-0.21**	0	-0.21**	
**Meaningfulness of Life**	0.17**	-0.005	0.165**	
**Responsibility**	-0.14**	0	-0.14**	

* P<0.05

** P<0.01

## Discussion

One of the possible explanations about the positive relationship between perceived stress with anxiety and depression symptoms can be traced back to sociocultural factors. Based on these factors, anxiety and depression are responses to environmental stress factors (43). As much as the environmental stress is perceived by a person, he/she will experience stress and lack of security against threats, so his/her self-esteem would be affected accordingly. If the self-esteem is low, the person will be prone to anxiety and depression in stressful situations (44).

Another finding of this study was the positive relationship between death anxiety and loneliness with the perceived stress. Death and loneliness are representing human instability in life (7). On the other hand, instability leads to stress (45). 

Moreover, findings of this study revealed a negative relationship between responsibility and meaningfulness of life with perceived stress. In addition, humans need a meaningful world and absurdity that arise from freedom is not desirable(6,7). Thus, if a person makes decisions only based on the personal criteria he or she has created without considering the lack of external criteria and is held accountable for those decisions that were made based on a meaningless world, he will develop a real relationship with the world and he makes decisions and makes choices chooses based on limitations of life. As a result, he will not experience imbalance between environmental demands and his capabilities, and thus he will not perceive stress and pressure.

By examining the model it was revealed that perceived stress plays a mediating role in the prediction of anxiety based on facing the existential issues. The literature review showed that studying this model had been completely neglected in the past empirical researches and it is novel in terms of experimental investigation. According to this finding, the theory of existentialism is able to analyze the development of psychopathology. It seems that facing the existential issues represents an imbalance between individuals’ abilities and demands of environment, which means existential issues can be perceived as stress, and perceived stress can lead to psychological trauma including anxiety. 

The results indicated that a part of the research model about the mediating role of perceived stress in the relationship between facing the ultimate concerns and symptoms of depression was not confirmed. The findings of the studies (23, 46) that assessed the effect of mediating role of perceived stress in the relationship between depression and meaninglessness and freedom were not in line with the findings of the present study. One of the possible explanations for the indicated findings is the incidence of depressive disorders in the spectrum of anxiety disorders (1). Therefore, researchers can investigate the depression symptoms as a product of anxiety symptoms, which means anxiety and depression symptoms may have a causal relationship. Moreover, according to the study participants who were adult students, another possible explanation also raises. Since anxiety typically begins at a younger age than depression, the lower mean age of the present sample (21.48) may explain the lack of meaningfulness of the research model on depression symptoms (1,20) and at the same time its significance for anxiety symptoms.

## Limitation

The sample was limited to Ferdowsi University of Mashhad in 2017-18. This limit should be considered in the generalizability of the results. Another limitation of this study was its retrospective self-report method, which means that participants' responses may have been affected by current events and that they might have not reflected their usual conditions. The third limitation of the present study was the correlational design that could cause limitations in the causal interpretations. The most important limitation of this study was the lack of full compliance with the characteristics of experimental studies with existential issues (7).

## Conclusion

This study reviewed the contributing factors that create anxiety symptoms from a new angle. This study revealed that facing existential issues through the mediating role of perceived stress engenders symptoms of anxiety. Therefore, education and health programs could be designed to prevent and treat anxiety. In doing so, the individual should be able to manage the perceived stress by focusing on how to face the existential issues to reduce anxiety. Solutions based on existential concepts can be useful to prevent and to treat anxiety. These solutions can be useful, specifically in those who do not respond to the prevention and treatment methods based on cognitive behavior. 

## References

[1] Craske MG (2012 Sep). Transdiagnostic treatment for anxiety and depression. Depression and anxiety.

[2] Motevalian S, Amin Esmaeili M, Hajebi M, Hefazi A, Rad Goudarzi R, Rahimi Monaghar A, Sharifi V (2014).

[3] Leventhal AM, Zvolensky MJ (2015). Anxiety, depression, and cigarette smoking: a transdiagnostic vulnerability framework to understanding emotion-smoking comorbidity. Psychol Bull.

[4] Bishop SJ, Gagne C (2018). Anxiety, depression, and decision making: a computational perspective. Annu Rev Neurosci.

[5] Craske MG, Wolitzky-Taylor KB, Mineka S, Zinbarg R, Waters AM, Vrshek-Schallhorn S (2012). Elevated responding to safe conditions as a specific risk factor for anxiety versus depressive disorders: evidence from a longitudinal investigation. J Abnorm Psychol.

[6] McElroy E, Fearon P, Belsky J, Fonagy P, Patalay P (2018). Networks of Depression and Anxiety Symptoms Across Development. J Am Acad Child Adolesc Psychiatry.

[7] Zafirides P, Markman KD, Proulx T, Lindberg MJ (2013). Psychotherapy and the restoration of meaning: Existential philosophy in clinical practice.

[8] Walsh SJ, Yalom, Irvin (1980). Existential Psychotherapy.

[9] Schneider KJ, editor (2011). Existential-integrative psychotherapy: Guideposts to the core of practice.

[10] Becker E (1973). The denial of death.

[11] Boss M (1977). Existential foundations of medicine & psychology.

[12] Ozanne AO, Graneheim UH, Strang S (2013). Finding meaning despite anxiety over life and death in amyotrophic lateral sclerosis patients. Journal of clinical nursing.

[13] Coulehan J (2018). Suffering, hope, and healing. InHandbook of pain and palliative care.

[14] Fava M, Rush AJ (2006). Current status of augmentation and combination treatments for major depressive disorder: a literature review and a proposal for a novel approach to improve practice. Psychotherapy and psychosomatics.

[15] Stålsett G, Gude T, Rønnestad MH, Monsen JT (2012). Existential dynamic therapy (“VITA”) for treatment-resistant depression with Cluster C disorder: matched comparison to treatment as usual. Psychother Res.

[16] Lazarus RS (1966). Psychological stress and the coping process.

[17] Yew SH, Lim KM, Haw YX, Gan SK (2015). The association between perceived stress, life satisfaction, optimism, and physical health in the Singapore Asian context. Asian Journal of Humanities and Social Sciences (AJHSS).

[18] Cohen S, Kamarck T, Mermelstein R (1983 ). A global measure of perceived stress. J Health Soc Behav.

[19] Pereira-Morales AJ, Adan A, Forero DA (2019). Perceived stress as a mediator of the relationship between neuroticism and depression and anxiety symptoms. Current Psychology.

[20] Association AP (2013). Diagnostic and statistical manual of mental disorders (DSM-5®).

[21] Ruscio AM, Gentes EL, Jones JD, Hallion LS, Coleman ES, Swendsen J (2015). Rumination predicts heightened responding to stressful life events in major depressive disorder and generalized anxiety disorder. J Abnorm Psychol.

[22] Siddaway AP, Taylor PJ, Wood AM, Schulz J (2015). A meta-analysis of perceptions of defeat and entrapment in depression, anxiety problems, posttraumatic stress disorder, and suicidality. Journal of affective disorders.

[23] Ventegodt S, Kandel I, Neikrug S, Merrick J (2005). Clinical holistic medicine: the existential crisis--life crisis, stress, and burnout. ScientificWorldJournal.

[24] Pines AM (2017). Burnout: An existential perspective.

[25] Ekedahl M, Wengström Y (2007). Nurses in cancer care—stress when encountering existential issues. Eur J Oncol Nurs.

[26] Antony MM, Bieling PJ, Cox BJ, Enns MW, Swinson RP (1998). Psychometric properties of the 42-item and 21-item versions of the Depression Anxiety Stress Scales in clinical groups and a community sample. Psychological assessment.

[27] Krejcie RV, Morgan DW (1970). Determining sample size for research activities. Educ Psychol Meas.

[28] Besharat MA (2005). Evaluating the Depression Anxiety Stress Scale (DASS-21) in clinical samples and the general population. Research report.

[29] Daza P, Novy DM, Stanley MA, Averill P (2002). The depression anxiety stress scale-21: Spanish translation and validation with a Hispanic sample. Journal of Psychopathology and Behavioral Assessment.

[30] Lovibond PF, Lovibond SH (1995). The structure of negative emotional states: Comparison of the Depression Anxiety Stress Scales (DASS) with the Beck Depression and Anxiety Inventories. Behaviour research and therapy.

[31] Norton PJ (2007). Depression Anxiety and Stress Scales (DASS-21): Psychometric analysis across four racial groups. Anxiety Stress Coping.

[32] Templer DI (1970). The construction and validation of a death anxiety scale. J Gen Psychol.

[33] Rajabi Gh, Bohrani M (2002). Factor analysis of Death Anxiety Scale questions. Journal of Psychology.

[34] Tavakoli MA, Ahmadzadeh B (2011). Investigation of Validity and Reliability of Templer Death Anxiety Scale. Thought & Behavior in Clinical Psychology.

[35] Russell DW (1996). UCLA Loneliness Scale (Version 3): Reliability, validity, and factor structure. J Pers Assess.

[36] Bohayraei H, Deavar A, Ahadi H (2010). Loneliness Scale Standardization, third edition (UCLA) in the community of students in Tehran. Applied Psychology.

[37] Steger MF, Frazier P, Oishi S, Kaler M (2006). The meaning in life questionnaire: Assessing the presence of and search for meaning in life. J Couns Psychol.

[38] Eshtad E (2009). Study the effectiveness of cognitive group therapy on subjective well-being. Thesis Master of Clinical Psychology.

[39] Groth-Marnat G, Pasha-Sharifi H, Nikkhoo M (2008). Handbook of psycholgical assessment: for clinical psychologists, counselors and psychiatrists.

[40] Gough HG (1987). California psychological inventory: Administrator's guide.

[41] Atef-Vahid MK, Nasr-Esfahani M, Fethullah P, Shojaei M (2005). Standardization of the California Psychological Inventory. Journal of Psychiatry and Clinical Psychology.

[42] Pasha Gh, Goudarzian M (2008). The relationship between identity styles and moral development of students' personal responsibility. New findings in Psychology.

[43] Safai m, Shokri A (2014). Stress Assessment in cancer patients, the validity of the Perceived Stress Scale factor in Iran. Psychiatric Nursing.

[44] Sharf RS (2015). Theories of psychotherapy & counseling: Concepts and cases.

[45] Thoits PA (2013). Self, identity, stress, and mental health. Handbook of the sociology of mental health.

[46] Mascaro N, Rosen DH (2006). The role of existential meaning as a buffer against stress. Journal of Humanistic Psychology.

